# Molecular Techniques for the Detection of Organisms in Aquatic Environments, with Emphasis on Harmful Algal Bloom Species

**DOI:** 10.3390/s17051184

**Published:** 2017-05-22

**Authors:** Linda K. Medlin, Jahir Orozco

**Affiliations:** 1 Marine Biological Association of the UK, The Citadel, Plymouth PL1 2PB, UK; 2 Max Planck Tandem Group in Nanobioengineering, Universidad de Antioquia, Complejo Ruta N, Calle 67, N° 52-20, Medellín 050010, Colombia; grupotandem.nanobioe@udea.edu.co

**Keywords:** molecular techniques, aquatic ecosystems, harmful algae bloom, FISH, sandwich hybridization assay, PCR, lab-on-a-chip, next generation system, isothermal amplification, hybridization chain reaction

## Abstract

Molecular techniques to detect organisms in aquatic ecosystems are being gradually considered as an attractive alternative to standard laboratory methods. They offer faster and more accurate means of detecting and monitoring species, with respect to their traditional homologues based on culture and microscopic counting. Molecular techniques are particularly attractive when multiple species need to be detected and/or are in very low abundance. This paper reviews molecular techniques based on whole cells, such as microscope-based enumeration and Fluorescence In-Situ Hybridization (FISH) and molecular cell-free formats, such as sandwich hybridization assay (SHA), biosensors, microarrays, quantitative polymerase chain reaction (qPCR) and real time PCR (RT-PCR). Those that combine one or several laboratory functions into a single integrated system (lab-on-a-chip) and techniques that generate a much higher throughput data, such as next-generation systems (NGS), were also reviewed. We also included some other approaches that enhance the performance of molecular techniques. For instance, nano-bioengineered probes and platforms, pre-concentration and magnetic separation systems, and solid-phase hybridization offer highly pre-concentration capabilities. Isothermal amplification and hybridization chain reaction (HCR) improve hybridization and amplification techniques. Finally, we presented a study case of field remote sensing of harmful algal blooms (HABs), the only example of real time monitoring, and close the discussion with future directions and concluding remarks.

## 1. Introduction

Molecular methods used to detect organisms are faster and more accurate than traditional methods, involving culture methods or microscopy. They are the preferred method of detection not only because of the increasing realization of the abundance of cryptic species that cannot be differentiated by any morphological means and the large number of micro-organisms that cannot be grown under laboratory conditions and thus go undetected but also because of the extensive training required to distinguish morphologically similar species. Molecular techniques are now used for identifying all organisms from vertebrates to viruses in a wide variety of programs [1].

The most efficient and effective way to characterize complex microbial samples is to use the small-subunit (SSU) and large sub-unit (LSU) ribosomal RNA (rRNA) genes [2], which have become the gold standards because a broader view of community structure and composition can be obtained by direct cloning and sequencing of these genes from natural samples [3]. Such methods have led to the discovery of an enormous amount of hidden biodiversity [4]. Probes (barcodes) can be designed from the RNA databases from Domain or other higher taxa down to a species [5,6,7]. The rRNA databases continue to increase in size and scope because these genes are routinely used for species identification. Species-specific probes designed from these genes can be applied for the analysis of any community and they can be detected using whole cell methods in which the cell remains intact (e.g., FISH) and thus also the morphology, or using cell free methods in which total nucleic acids are extracted and probes applied directly to the nucleic acid target (e.g., SHA, microarrays and biosensors). For some groups, such as the marine phytoplankton, detailed protocols can be found in the UNESCO manual for quantitative phytoplankton analysis edited by Karlsen et al. [8] and for microarrays in Lewis et al. [9]. One limitation of all of the methods presented below is if they are used to detect the species, they may not be able to predict if it is harmful. Whereas eukaryotic algae normally have toxic and non-toxic species, bacteria and cyanobacteria have toxic and non-toxic strains of the same species, species detection methods are not so useful. Some of the methods described can be applied to detect toxin genes in addition to identifying the species, which will identify the potential of the water body being investigated to become toxic.

## 2. Molecular—Whole Cell Methods

Fluorescence In-Situ Hybridization (FISH) refers to the application of an oligonucleotide probe bound to a fluorescent marker that penetrates a cell and hybridizes to the ribosomes inside the cell. The entire cell fluoresces a bright color because of the high target number of ribosomes in the cells of interest. The cell stays intact and co-occurring species can be discriminated when counterstained with an overall DNA stain, e.g., DAPI. Using this method any target organism can be easily identified at the light miccroscope level using fluorescent microscopy or by flow cytometry. Amann [10] was the first to show the detection of different species and even closely related, morphologically similar species or strains can be separated. This method when applied to plant material is challenging because plant material contains chlorophyll that naturally fluoresces. Fluoresceinisothiocyanate (FITC), one of the most commonly used fluorochromes as a marker on the probes (barcodes) fluoresces green, can be easily distinguished from the orange auto-fluorescence of the cell’s chlorophyll, whereas the red fluorescence of CY5-labeled probes can be more difficult to distinguish from the chlorophyll orange fluorescence, unless stronger bleaching compounds, dimethylformamide, are used to remove the chlorophyll [11]. FISH has been successfully applied for the detection of Harmful Algae as well as other algal groups [6,11,12,13,14,15,16,17] and to a wide variety of bacteria [10].

In the marine phytoplankton, many different types of cell walls and membranes occur, which creates a challenge to develop a FISH protocol capable of fixing all kinds of algal cells. Many naked cells rupture with some fixatives [18]. The saline ethanol method originally developed by Scholin and co-workers [19,20,21] used probes with more than two mismatches between target and non-target sequences. The saline ethanol fixative also extracts the chlorophyll from the cells and bleaches them, thus permitting better visualization of probe signals [19,20,21]. Groben and Medlin [10] found Scholin’s conditions not to be sufficiently stringent for a wide range of species and insufficient to distinguish single base mismatches between target and non-target. They developed a protocol that could be used with the widest range of phytoplankton cells from the most delicate to the most rigid while maintaining the stringency needed to discriminate single base mismatches. Formamide was added to the hybridization buffer and in the last washing step, the salt concentration was reduced to make the hybridization more stringent. Formamide concentrations, used to reduce the hybridization temperature to one that does not destroy the cell’s integrity, must be empirically determined for each probe. Sodium dodecylsulfate (SDS), commonly used in hybridization buffers, lyses the membranes of more fragile, naked cells. IGEPAL-CA630 (or the chemically identical NONIDET-P40) maintains cell stability permitting efficient probe penetration.

After FISH hybridization the search for cells and their enumeration with epifluorescence microscopy can be time consuming and susceptible to human error because of the variability in RNA content, which can cause weak signals or because cells are hidden under debris. Therefore, if many samples are to be analyzed, automated counting is preferred and flow cytometry (FCM) is a suitable alternative. Both liquid and solid phase cytometers (LFC/SPC) are available tools.

As cells in suspension pass through a narrow laser in single file, LFC measures the size of and counts them by their optical characteristics [22]. Cell identification and counting can be enhanced with the addition of FISH probes for greater differentiation of phytoplankton populations [23]. FISH for LFC has to be performed in suspension, which involves the cells being fixed in a tube, then centrifuged, then resuspended, repeatedly, for the various stages of the FISH protocol, which can result in high cell loss during these stages. If the tubes are treated with surfactants, then this problem can be remedied [24].

**Figure 1:**
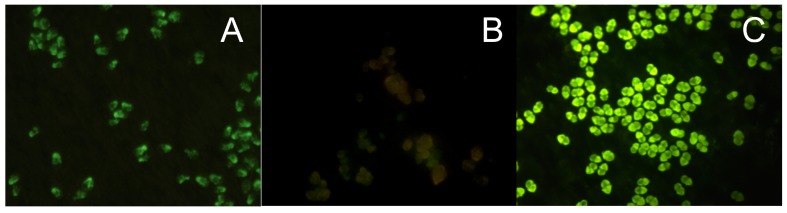
A comparison of FISH and Card FISH using the toxic dinoflagellate *Azadinium* spp. (**A**) FISH with a FITC label; (**B**) no probe control; (**C**) Card FISH FITC enhancement label.

Often the fluorescence signal of FITC-labeled bound probe can be too low for detection because of high auto-fluorescence of the chlorophyll in pigmented cells and because of low target number from a pico-sized cell or from a senescent cell (especially prokaryotic cells), and therefore a lower cellular ribosome content. Poor accessibility of the probe target sites in the rRNA molecule because of its secondary structure formation or because ribosomal proteins, which can block or cover probe-binding sites probe penetration [25], can be additional reasons for a low fluorescence yield. In these cases, an amplification of the fluorescent signal is required, such as the tyramide signal amplification (TSA) or the catalyzed reporter deposition (CARD) method, which is an enzyme catalyzed enhancement method of fluorescence signals. Horseradish peroxidase (HRP), linked to the 5’-end of an oligonucleotide probe and in the presence of small amounts of hydrogen peroxide converts its labeled substrate, tyramide, into short-lived, extremely reactive intermediates, which can be fluorescently detected [26,27,28]. These activated tyramides rapidly bind covalently to electron rich regions of adjacent proteins, such as tyrosines, only at or adjacent to the probe target sites where the HRP-labeled oligonucleotide probe is bound to its target [26,27,28]. Thus, the labeled tyramides are deposited multiple times at the hybridization site to achieve an enhanced signal [28] (Figure 1).

An FITC, Cy5, or Alexa fluorochrome can be bound to the tyramide, thus providing a series of labels with different excitation and emission wavelength [23]. Far greater fluorescence intensity, up to 20 times, can be obtained from this indirect labeling method than with a direct label [26]. Species in low abundance or senescent cells in a sample can easily be detected. Higher formamide concentrations must be used with the target probe to ensure probe specificity because the CARD FISH hybridization, which is an enzymatic reaction, must be performed between 35 and 37 °C. The tyramide signal amplification system has been successfully used to detect bacteria [27], cyanobacteria [26,29] picoplankton cells by fluorescent microscopy [30] and in flow cytometry [24], and bacteria associated with microalgae [31,32].

In contrast to LFC where the cells in liquid suspension are moved in single file through a stationary laser for excitation, in SPC, the laser is moved over immobilized cells on a membrane support [33]. The ChemScan system (Chemunex, Ivry, France) is a SPC for the detection and enumeration of fluorescently labeled microorganisms on filter membranes [34,35]. The ChemScan was initially developed for the fast detection of microorganisms in filterable products in industrial and environmental Microbiology, and has been optimized for Microbiological applications with standardized protocols [33]. It has recently been used for the detection of fluorescently labeled toxic microalgae with antibodies [36] and by FISH probes [37,38] and bacteria with FISH probes [39]. SPC is the only method with a detection limit of one cell per sample [40]. It has the advantage of combining LFC and image analysis [41] to allow a rapid enumeration of thousands of cells with similar accuracy to LFC [42]. CARD FISH is required for the ChemScan because normal FISH is not sensitive enough for the laser detection [37]. The only disadvantage of SPC counting is that it cannot distinguish long filamentous cells. It performs best with round and spherical cells. A validation of the positive counted cells is recommended, as the filter is transfered to a fluorescent microscope and each positive signal is verified before total automation can be reliable.

All FISH methods are limited by the number of species that can be detected under each detection method and within one experiment. Presently only two different kinds of fluorochromes, FITC and CY5, are routinely used for detection. LFC and SFC are also expensive tools for routine monitoring as is the synthesis of HRP-labeled probes. Monoclonal antibodies (MAbs) and polyclonal antibodies can detect cultured and field-collected cells and target to cell surface antigens [43]. Immunomagnetic beads coupled to both MAbs and polyclonal antibodies can achieve separation of target cells from mixed assemblages [44]. MAbs typically require development of hybridoma cell lines produced by fusing myeloma cells with spleen cells of mice that are immunized with the target antigen and are considerably more difficult and technically demanding to produce than polyclonal antibodies. MAbs are unlimited in supply because the hybridomas are immortal. No cell permeabilization is required as in FISH methods, and the fluorescence intensity is usually far greater than that of DNA probes and less affected by the cell’s physiological state [45].

## 3. Molecular—Cell-Free Format

All cell morphology is lost, if total nucleic acids are extracted from samples. Also free DNA from dead cells is extracted at the same time. Cell numbers from whole cell methods are often lower than those inferred from cell free methods and low rRNA content and free DNA have been cited as the cause of such discrepancies [46]. However, despite these discrepancies, several methods have been used that rely on high quality DNA or RNA extracted from environmental samples and have been successfully used to detect organisms from many different water types.

### 3.1. Sandwich Hybridization Assay (SHA)

**Figure 2:**
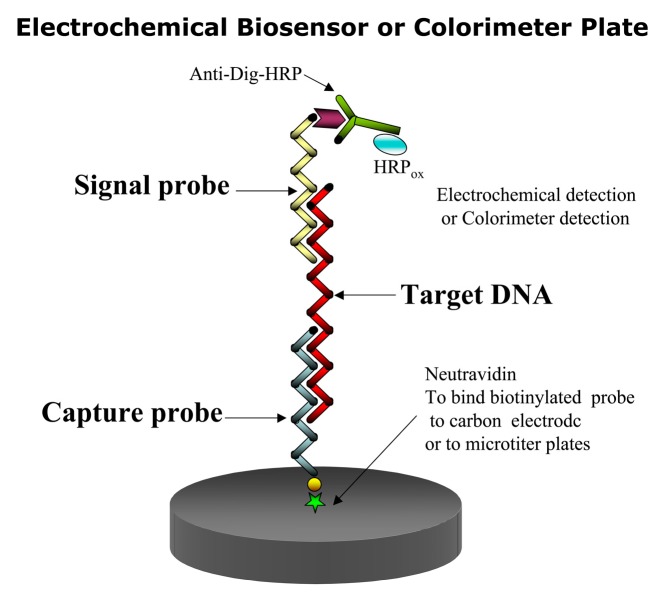
Scheme of a SHA hybridization. Capture probe is immobilized onto a carbon electrode, which then captures the target RNA, which binds to the signal probe with the DIG label to initiate the electro-chemical reaction.

In this assay, a capture and a signal probe bind the target DNA or RNA in the so-called sandwich hybridization (Figure 2). Thus two hybridization events are involved. In the first event the immobilized capture probe binds to the target sequence binds event, and in the second hybridization event, a signal probe linked to a recorder molecule [47,48], such as a fluorochrome or digoxigenin (DIG) binds to the first complex to facilitate its detection. To detect the target species, only one of the two probes need be specific. A capture probe can be immobilized on either a membrane, an electrode or a microtiter plate [49,50,51,52]. In the case of DIG, an antiDIG antibody is used for coupling a horseradish peroxidase (HRP) enzyme to the signal probe to form the final complex for signal amplification. HRP converts inactive substrates to a product that can be detected electrochemically or colorimetrically. The colorimetric SHA offers the cheapest and fastest way to test the specificity of the signal and capture probes [53].

SHAs have the advantage of being ultra-sensitive. This format maximizes discrimination of target from non-target sequences. Purification of target molecules (e.g., RNA) is not required [21]. The SHA method has been widely used for the detection of toxic algae [21,50,54] and has been formatted for an automated Universal Assay Processor (Saigene Biotech, Inc., Denver, CO, USA) that provides users with flexibility and control over various assay parameters (e.g., sequence, duration, and temperature of individual steps [55].

### 3.2. Biosensors

Biosensers are simple, fast and can be manufactured into compact, inexpensive devices [49,50,51,52,53,54,55]. They can overcome limitations caused by traditional detection and subsequent quantification. Among the detection methods applicable to biosensors, electrochemical detection enjoys from high sensitivity and selectivity and rapid response. Therefore, it has low power requirements, which makes this method more versatile and amenable for monitoring in outside settings. Electrochemical sensors can detect nucleic acids directly in complex environemental samples, which gives them a valuable advantage over other molecular methods, such as PCR, which requires target purification and amplification [56] and is sensitive to enzyme inhibitors. Biosensors are powerful tools for species detection. Among them, those based on the combination of the SHA method with electrochemical detection of bound nucleic acid target molecules have proven to be the most successful [49,50,51,52]. Diercks et al. [53] demonstrated that this detection system could be adapted into a multiprobe biosensor for its use in a semi-automated device for the simultaneous detection of 14 target species of toxic algae. All steps needed to elucidate the different steps of the biosensor fabrication process from the electrochemical point of view, proof of concept with different algal species, and the evaluation of the influence of the transducer platform geometry and material in the biosensor analytical performance [49,50]. All components of the electrochemical biosensor SHA assay have been optimized with calibration curves for 14 toxic algal species [52].

Fiber-optic genosensors for toxic dinoflagellates have been introduced as another type of biosensor that [57,58] employs a SHA detection system. The capture probes are placed on microspheres at the end of each optical fiber to capture the rRNA of the HAB species. After hybridization, the microarray is dipped into formamide to denature the capture RNA and its signal probe for its reuse. It could detect as few as five cells in a mixed phytoplankton sample.

The biosensor SHA assay can also be used with colorimeter detection. With a different substrate, the anti-digoxigenin antibody conjugated to HRP can produce a colored product whose intensity can be measured in a spectrophotometer or photographed by a camera, thus becoming a chemiluminescent biosensor.

### 3.3. Microarrays

**Figure 3:**
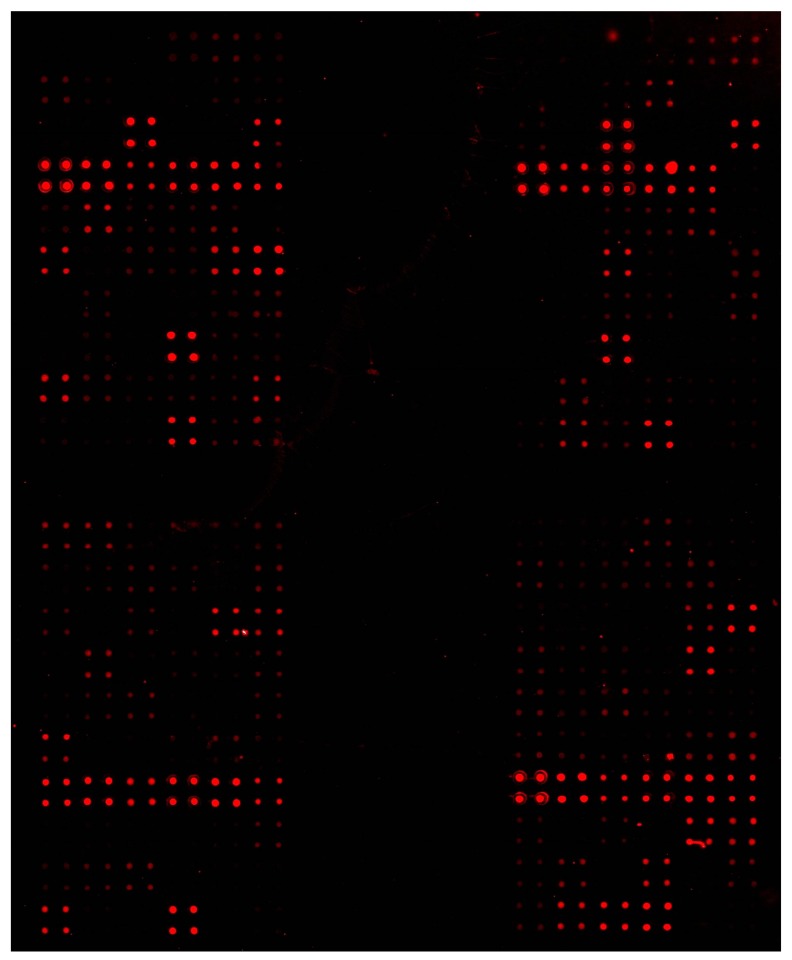
Image of a scanned DNA-microarray from a field sample taken in the MIDTAL project for the detection of toxin algae. Each cluster of four dots represents replicate probes specific for one species spotted onto the glass slide and hybridized to fluorescently labelled RNA from the field sample.

A microarray consists of DNA sequences (barcodes) that are applied to the surface of a glass slide with special surface properties in an ordered array similar to a dot blot [59,60,61,62] (Figure 3). Thus, the ability to detect potentially thousands of targets in one hybridization experiment makes the microarray detection system one of the most powerful molecular tools available today, when targets are known. Microarray production, nucleic acid isolation and preparation, hybridization, and data analysis are the required steps in a microarray experiment. The target nucleic acids are labeled with a fluorescent dye, which can be incorporated directly to the nucleic acid or via indirect labeling of other substances [61,62,63]. Then the labeled targets are hybridized to the probes on the microarray. The laser in a microarray scanner scans the slides and the hybridization pattern captured via fluorescent excitation indicates which species are present [60]. DNA microarrays, or phylochips as they have been termed, have been used to identify phytoplankton [63], toxic algae [64,65,66,67,68,69,70,71,72,73,74,75,76,77], bacteria [78,79,80,81,82,83,84], and eggs and larvae from fish species [85]. Phylochip^®^, a universal microarray for all prokaryotic organisms is commercially available and circumvents the long analysis time to perform community analysis for the prokaryotes using other molecular tools. Microarray analysis of environmental samples has now received an ISO number (ISO 16578, 2013(en)) and thus is now a fully accredited method for determining the concentration of DNA in any environmental sample.

The EU project, MIDTAL [86], was devoted to the construction of a universal microarray for the detection of toxic algae. The MIDTAL project produced a standardized method of hybridization, analysis and calibration [9] to convert the signal to cell numbers for the monitoring of toxic algae. This is essential for monitoring because nearly all decisions on fisheries closure are based on cell numbers that trigger toxicity testing. This microarray was field tested for 2 years in five EU countries that regularly monitor for toxic algae showing good correlations with standard cell counting methods.

A subsequent EU project, μAQUA, was devoted to the construction of a universal microarray for the detection of freshwater pathogens and to development of novel tools for toxin detection by cyanobacteria. This project was field tested also for two years in 8 EU countries and the results from some of these studies have been published [87,88]. Among the novel tools developed for toxin detection, a toxin array was dedicated to the detection of the messenger RNA from cyanobacterial toxin genes being expressed. This microarray incorporated a reverse transcriptase elongation of the probe (barcode) used to capture the messenger RNA expressed from the toxin genes and this elongation incorporated fluorescent nucleotides which functioned to boost the signal on the microarray several times above the background level [89]. Thus, the messenger RNA that was being expressed in very low quantities, that was below the detection level of standard HPLC methods, could now be detected. This method could serve as an early warning system showing the potential of any water body to become toxic.

A different type of multiplex system, the Luminex system, uses principles of either quantitative fluorescent microscopy or fluorescent flow cytometry to enable simultaneous identification. Each unique population of coded beads is dyed internally with a different ratio of two fluorophores and covalently functionalized with a species-specific capture probe that binds biotinylated target DNA. Hybridization of the target is detected using a reporter molecule (e.g., phycoerythrin coupled to streptavidin). Multiplexed, bead-based arrays that employ flow cytometric detection of color-coded fluorescent bead populations have been developed as a microarray for toxic algal species [90,91]. Luminex-based detection strategies are still considered preliminary research and development efforts.

### 3.4. qPCR

**Figure 4:**
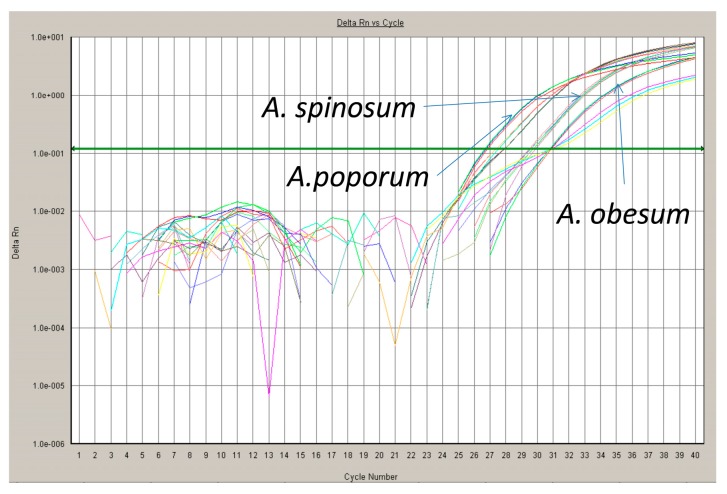
Typical cycle of a qPCR reaction, field sample separating three toxic species of the dinoflagellate *Azadinium*, courtesy of Dr. K. Toëbe.

One of the most powerful technologies in molecular biology is the polymerase chain reaction (PCR) [92]. There is no information about the quantity of starting material in the samples analyzed if traditional qualitative “endpoint” PCR is used. However, in qPCR, information about the quantity of starting material can be calculated because by using fluorescent markers that are incorporated into each PCR product as amplification proceeds, data can be collected over each PCR cycle. The change in fluorescence that is measured as PCR labeled amplicons are accumulated in each cycle is directly proportional to the amount of starting material (Figure 4). These data are monitored with an integrated detection system during the linear exponential phase of the PCR [92]. Closely related species or populations can be distinguished because qPCR can distinguish base pair differences. External standards for quantifying the amplified DNA must be measured if environmental samples are to be analyzed. This could be a dilution of plasmids or DNA derived from laboratory cultures with a known analyze concentration of the target template. A standard curve must be made for each target species to infer its concentrations in an unknown sample because of differences in DNA content per cell [93]. Copy number of the rDNA genes may vary among different strains of an organism/species and should be considered in calculating the starting concentration of any target [94]. Copy number can be inferred from qPCR experiments.

In the SYBR Green approach, the fluorescent dye, SYBR Green, binds to the minor groove of double stranded DNA (dsDNA). The increase in fluorescent emission is proportional to an increase in the PCR-amplified dsDNA during each cycle. In the method, primer-dimers are counted as amplified DNA because of the unspecific binding of SYBR Green to all dsDNAs generated during the PCR cycles. Thus to avoid primer-dimer artifacts, critical primer design is needed. They can be identified by their lower melting temperature compared to that of the target amplicon and are revealed by performing melting curve analyses [95].

In the TaqMan, molecular beacon and hybridization probe assays, a specific fluorigenic oligonucleotide probe is used with specific or non-specific primers. When the specific probe binds to its target, there is a transfer of energy from an excited fluorophore, the donor, to another fluorophore, the acceptor [96]. This enhanced fluorescence is termed fluorescence resonance energy transfer (FRET). A rapid and quantitative enumeration of several organisms within one sample (multiplex PCR) can be achieved through the use of specific primers and oligonucleotide probes, labeled with unique fluorescent dyes with different excitation wavelength. The number of detectable target genes in one sample is limited by the number of available fluorescence reporter dyes for the separate probes. A limit of six species to be detected in one sample is a general rule. Multiplex qPCR experiments require elaborate adaptations and have to be carefully optimized [97].

Digital (d)PCR is another method that is gaining popularity [98]. A sample is dispersed as an emulsion into micro-well plates so that a single droplet contains ≤1 template molecules. There are thousands of droplets in each well. One sample will be partitioned into the droplets available. Sample partitioning permits an estimation of the number of template molecules by assuming that the population of molecules follows a Poisson distribution. Thus, each part will contain either “0” or “1” molecules, or a negative or positive reaction, respectively. Genomic DNA is fragmented using DNaseI to produce 2–4 kb fragments as template. The template mixture is partitioned into droplets and paired with primer pair droplets, both of which enter a microfluidic chip at a rate of about 3000 droplets per second. The primer pair droplets are smaller than the template droplets and move through the channels faster until they contact the preceding template droplet. Field-induced coalescence of these droplet pairs results in the two droplets merging into a single PCR droplet, which is collected and processed as an emulsion PCR reaction [99]. After PCR amplification, nucleic acids may be quantified by counting the wells that contain PCR end-products as positive reactions. To improve the diversity of the assay, different primer combinations can be allocated into the different wells of the plate. There are currently about six different platforms for digital PCR but basically fall into two categories: chip-based and droplet based [100]. The microfluidic-chip-based dPCR can have up to several hundred partitions per panel, whereas droplet-based dPCR usually has approximately 20,000 partitioned droplets and can have up to 10,000,000 per reaction. The total number of analyzed partitions and partition volume depends on which dPCR platform used for the measurement. Te et al. compared qPCR and dPCR to estimate the simultaneous quantification of *Microcystis* and *Cylindrospermopsis* [101]. The former was found easier to use but the latter was more sensitive and thus more accurate.

Different DNA extractions are known to yield different amounts depending on the extraction method used. Also humic substances are known to inhibit PCR reactions. These potential drawbacks and limitations of qPCR problems can be resolved or minimized by using a high quality DNA isolation method, such as TriReagent. qPCR can be easily performed immediately after in-situ sampling onboard ship or on shore. Preserved samples can also be used but these preservatives may cause PCR inhibition. The preferred strategies are no preservation, or preservation using ethanol, coupled with freezing. Hosoi-Tanabe and Sako [102] could detect and quantify target cells after three years from field samples processed in this way. The sensitivity of qPCR is lowered with samples preserved with formalin and glutaraldehyde. Lugol’s iodine, a commonly used fixative has been reported to lower the sensitivity of some qPCR experiments [102], but successfully applied in others that detected toxic algae [103,104]. Multiplex qPCR experiments require extensive optimizations to make different primers and/or probes work together in a single PCR experiment. Handy et al. [93] compared multiplexing vs single probe PCR and found that although both methods were sensitive and effective, multiplexing was more efficient once optimized.

### 3.5. RT-qPCR

When the starting material in a qPCR experiment is RNA, this is termed quantitative reverse transcription PCR (RT-qPCR). Total RNA or messenger RNA (mRNA) is first transcribed into complementary DNA (cDNA) by reverse transcriptase, which is used as template in the qPCR reaction. RT-qPCR can be performed in one or two steps [105]. One-step assays combine the RT step and qPCR step in a single reaction tube along with buffers needed for both reactions. Target-specific primers are used in one-step RT-qPCR. In two-step assays, the RT and qPCR steps are performed separately, with different optimized buffers, reaction conditions, and priming strategies. It is almost impossible to optimize the one-step method because both reactions are very different from one another, requiring different temperatures, etc; thus being less sensitive. However, the one step method is more economical and less prone to pipetting errors. In the two-step method, the cDNA generated can be reused at a later date if needed. More different kinds of targets can be interrogated with the two-step method.

### 3.6. Lab-on-a-Chip

(LOC) is a device that integrates one or several laboratory functions into a single integrated system (the so-called a “chip”, hence lab-on-a-chip) of only millimeters to a few square centimeters in size to achieve automation and high-throughput screening or single or multiple targets [106,107]. Extremely small fluid volumes down to less than a few pico liters are easily handled by LOCs. Detection can be achieved fluorimetically, colorimetrically or electrochemically. They are usually designed to be single use and disposable and their applications range across a wide variety of disciplines [106]. This technique is an emerging technology with many companies offering custom designed LOCs. Some examples of developed LOCs include those for many pathogens to medical point of care [107,108].

### 3.7. Next-Generation Sequencing or High Throughput Sequencing (NGS or HTS)

Ebenezer et al. [109] summarized the NGS or HTS technologies available and their major features. Life science studies using molecular techniques, such as full genome sequencing (de novo sequencing and resequencing), amplicon sequencing, transcriptome sequencing, and metagenomics has employed NGS or HTS technologies. These techniques with pyrosequencing generate much higher throughput data, such that millions to billions of sequencing reactions take place in small reaction volumes at the same time. In field sample studies, NGS or HTS technologies gather DNA data from both environmental DNA and/or PCR products amplified from environmental DNA. Because DNA templates are bound to substrates and amplified by PCR to generate clonal representatives, NGS or HTS does not require cloning of template DNA into bacterial vectors. The number of sequence reads by the NGS or HTS methods are continually increasing with upwards of 500 bp reads, NGS or HTS is fast becoming the tool of choice for the identification and detection of microbes from environmental samples [109]. However, the long time to process data is still a major concern and makes the use of microarrays more attractive as a means of analyzing large numbers of samples when targets are known.

The dominance of NGS sequencing as performed by Illumina is being challenged by Oxford Nanopore [110]. A new type of “nanopore” DNA sequencer, the MinION, is now available. It analyzes DNA by drawing the molecules through a tiny, delicate pore in near real time. Each combination of the genetic letters A, G, C, T produces a change in electrical current as the DNA moves through the pore, allowing the molecule to be read (sequenced). The nanopore is small enough to be portable and reads out very long stretches of DNA up to more than 150 kb.

## 4. Enhancing Performance

A common concern in molecular techniques for the detection of organisms in aquatic environments is related to the sample complexity in terms of abundance and diversity of planktonic species. The question of wide types of species that cohabit in a certain aquatic ecosystem may be potentially solved by different strategies, such as multiplexed biosensors, microarrays and NGS, etc., as mentioned in the preceding section. However, some times the low abundance of some species in the environment is the critical factor that determines the feasibility of their monitoring. In this context, in this section we will review some approaches that have been used to enhance the performance of molecular techniques that go from development of nano-bioengineered platforms and the use of preconcentration systems to improved hybridization and amplification methods.

### 4.1. Nano-Bioengineered Probes and Platforms

Biosensors have gain importance for specie-specific detection tanks to their outstanding features in terms of simplicity, portability and miniaturization possibilities, along with those already mentioned in the previous section. Molecular techniques for monitoring aquatic organisms based on electrochemical biosensors are based on hybridization of specific nucleotides with their complementary strands, linked both to a solid support and to a reporter able to produce a signal in the presence (or not) of an electroactive indicator. The platform has the capability of transforming (transducing) the generated signal into an electrochemical signal easy to be recorded and interpreted. Electrochemical detection can be either direct, both by the intrinsic electro-activity of the nucleic acids and DNA duplex electroactive intercalators, or indirectly by means of electroactive probes [111]. The resultant signal can be amplified by coupling enzymes, fluorescent labels, inorganic nanoparticles, or through nanomaterial-based hybrids platforms or a combination of the above [112].

Stability, sensitivity, hybridization efficiency and minimization of specific adsorption can be modulated by controlling the surface chemistry and surface coverage. Mostly, noble metals and semiconductor materials such as carbonaceous materials and polymers are the materials that work as transducers. However, decoration of the transducers with nanomaterials, including nanoparticles, carbon nanotubes, graphene, quantum dots, etc, has caused a tremendous evolution of (bio)sensor devices [113]. The resultant nano-bioengineered structures are the responsible for the great enhancement in sensitivity that more recent genosensor-based approaches exhibit today. The higher sensitivity is related to the increased surface area and enhanced catalytic properties, among other improved features from the nanostructures. For instance, bioreceptors immobilized in an irregular nanostructured surface facilitates the accessibility of the target molecules and thus promote more efficient electron transfer processes and faster reaction kinetics. Nano-bioengineered probes can be either anchored at the transducer or coupled to reporters. In the pioneering work of Prof. Mirkin, the first rational assembly of nanoparticles into a macroscopic material using DNA strands appeared. Such a remarkable discovery made it possible to tailor the nanoparticles properties by means of the specificity of the DNA interaction [114]. More recently, Prof. Bard applied Pt nanoparticles for electrochemical amplification of DNA analysis by a single label response [115].

Thanks to the intrinsic electrochemical properties of AuNPs, they are the most common material selected for signal amplification of DNA-based electrochemical biosensors. Coupling of DNA sequences with nanoparticles allows for the development of genosensors of highly improved performance. Target concentration can be inferred either after dissolution of AuNPs in acidic media, with subsequent determination by anodic-stripping voltammetry [116], or by their coupling with electroactive complexes with further interrogation by chronocoulometry [117]. Gold tracer ‘amplification’ by silver deposition on the Au surface has also been applied to enhance sensitivity and lower the detection limits of these kind of biosensors [118]. Coming to aquatic ecosystems monitoring, a highly sensitive and specific gold-nanoparticle based electrochemical genosensor towards the 18S rRNA and internal transcribed spacer regions of the fish pathogen *Aphanomyces invadans* was shown to be suitable as a diagnostic tool in the aquaculture industry [119].

Inorganic and organic quantum dots (QDs) have demonstrated to be an alternative to enzyme-based amplification with potential to overcome its limitations in terms of long-term stability and storage as well as inhibition in harsh environments. For example, an electrochemical biosensor assay with QDs of PbS, CdS, ZnS as labels was highly sensitive and specific for simultaneous detection of non-protein coding RNA sequences of *Vibrio cholerae*, *Salmonella* sp. and *Shigella* sp.; with high potential for monitoring multiple pathogens in environmental samples [120].

Hybrid nanocomposites have shown enhanced properties in the development of molecular biosensors respect to the corresponding materials acting alone. For example, a new DNA biosensor based on reduced graphene oxide decorated Ceria nanoparticles was designed for sub-femtomolar detection of *Aeromonas hydrophila* DNA sequence in fish pond water [121]. A selective capture probe was immobilized at a graphene oxide (GO)-cerium nanocomposite and the Fast Fourier transform square wave voltammetry (FFT-SWV) was used to discriminate changes in target analyte concentrations in the presence or a redox marker. Amperometric detection of *Escherichia coli* O157, H7 was achieved by a GO, thionine (Thi), Au nanoparticles coated SiO_2_ nanocomposite (GO-Thi-Au@SiO_2_)-based tag [122]. Large amounts of signal DNA and G-quadruplex were immobilized on the nanocomposite, where hemin was further intercalated to obtain a hemin/G-quadruplex structure as HRP-mimicking DNAzyme. Although the assay was only tested in laboratory conditions, it holds promise for aquatic systems monitoring.

### 4.2. Pre-Concentration and Magnetic Separation Systems

Some pre-concentration systems have been proposed to solve the limitation of low abundance of microorganisms in aquatic ecosystems. The most common practice consists of pre-concentrating the microorganisms by filtering large volumes of water samples. This practice is time- and power-consuming and not amenable with monitoring in outside settings. However in the EU μAQUA project, >50 L of water were filtered using hollow fiber filters in relatively short amounts of time (ca. 30 min) and the filters effectively concentrated all organisms down to viruses in the 50 L into a one liter volume for easier down stream analysis [86,87].

Magnetic micro and nanocarriers have gained tremendous attention as pre-concentration systems because of rapidity, practicality and cost-effectiveness, as well as low workload, high speed and high-throughput automation [123]. Capture probe-modified magnetic particles are added to the target DNA (RNA) containing sample, which is selectively captured. The captured target can be easily confined in a specific place by a magnet and the sample washed off. Therefore, the resultant concentrated target is free of interferences that can eventually be present in the initial sample. An integrated microfluidic PCR system enabled pre-concentration of microbial pathogens by magnetic separation prior to DNA amplification by PCR [124]. The system combined the pre-concentration capabilities of magnetic nanoparticles with PCR amplification for the fast, specific, and quantitative detection of the microbial pathogens in samples of large volume. A sensitive and selective genomagnetic assay based on in-situ DNA amplification with magnetic primers was developed by DNA double-hybridization with both a digoxigenin probe and a biotinylated capture probe, with further binding to streptavidin-modified magnetic beads [125]. Although the DNA amplification-based genosening strategy was demonstrated for the electrochemical detection of food pathogens, it is promising for sensing a myriad of species including environmental pathogens from aquatic ecosystems.

Magnetic beads have been also introduced as a separation technique in diagnostic Microbiology since more than two decades. However, their application for monitoring environmental microorganisms is rare [126]. Nucleic acids can be sequenced by solid phase assistance, in which biotin-labeled amplicons from the target sequences are linked to streptavidin-coated magnetic beads. Denaturation can be achieved by treatment with NaOH or heat and the magnetic bead-linked and the free dissolved strands separated by a magnet. Both strands can be recovered for sequencing.

### 4.3. Solid-Phase Hybridization

The target DNA can be immobilized by linking it with a solid phase support to capture low-abundance target DNA sequences. It has shown to be a high-performance and high-throughput method to detect the presence of little amounts of target genetic material. For example, femtograms of ribosomal RNA from bacterial fish pathogens, including *Aeromonas salmonicida*; *Tenacibaculum maritimum*; *Lactococcus garvieae*; and *Yersinia ruckeri* were detected by solid phase PCR. The approach was based on a RT-PCR-enzyme hybridisation assay with NucleoLink(TM) strips for liquid- and solid-phase PCR in one tube. It detected 4 fg of rRNA from pure cultures and between 1 and 9 CFU per 200 µL sample volume from culture media [127].

### 4.4. Isothermal Amplification

Unlike PCR, isothermal amplification is a nucleic acid amplification technique where the reactions take place at a constant temperature, thus avoiding the thermal cycler requirement and making possible amplification in outside settings. Among isothermal amplification techniques, loop mediated isothermal amplification (LAMP) is a variant where the target sequence is amplified using either two or three sets of primers, and a polymerase with high strand displacement and replication activity. Additionally, a pair of “loop primers” can further accelerate the reaction. LAMP is considered an enhanced amplification method because the amount of DNA produced is considerably higher respect to that from normal PCR-based amplification. A rapid RT-LAMP assay was established for the highly sensitive and specific detection of *Eriocheir sinensis reovirus*, a pathogen that causes high mortality in crab aquaculture [128]. The assay was more cost-efficient and proper for field monitoring than the normal PCR amplification method. Variations of LAMP, such as multiple endonuclease restriction (MERT)-LAMP, have been successfully developed for simultaneous detection of *V. parahaemolyticus* and *V. vulnificus* strains in a single reaction. These two marine seafood-borne pathogens cause severe illnesses in humans and aquatic animals. The approach was sensitive, specific and rapid, and with potential for simultaneous screening of the pathogens in a wide variety of samples. A colorimetric LAMP assay was also evaluated for visual detection of *Streptococcus agalactiae* and *Streptococcus iniae* in *Tilapia* [129]. The method demonstrated usefulness for monitoring fish health in grow-out ponds, in the fish farming industry. Other pathogens from aquatic environments, such as *Edwardsiella tarda* [130], *Vibrio anguillarum* [131], *Vibrio vulnificus* [132], and *Lactococcus garvieae* [133], have been also detected by LAMP.

### 4.5. Hybridization Chain Reaction

**Figure 5:**
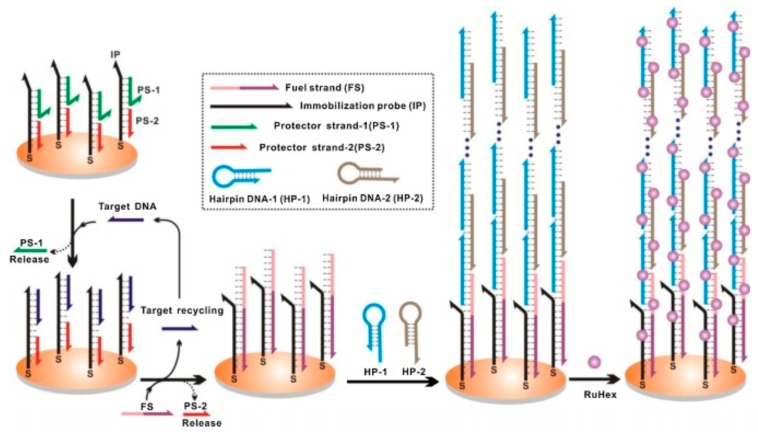
Tandem DNA-fueled target recycling and hybridization chain reaction concept. Reproduced with permission from [135].

Hybridization chain reaction (HCR) is a technique based on a chain reaction of recognition and hybridization events between two sets of stable DNA hairpin molecules that storage potential energy and offers an enzyme-free alternative for the rapid detection of specific DNA sequences [134]. Unlike normal PCR, in HCR, the binding of DNA to a substrate accomplishes not only recognition, but also linear signal amplification without any external input and at room temperature. In this technique, the DNA hairpin monomers coexist in solution until the target, acting as initiator strands, triggers a cascade of the hybridization events that yields double helices analogous to the alternating copolymers [134]. The technique has potential for highly efficient amplification of short sequence oligonucleotides. For example, detection of Bacteria, Archaea and Methanosaetaceae in an anaerobic sludge sample was achieved by simultaneous in-situ DNA-HCR [135]. Analogously, an improved in-situ DNA HCR named quick HCR-FISH was tested for the rapid and sensitive identification of marine bacteria with low rRNA contents not only in seawater but also in sediment samples [136]. Recently, HCR acting in tandem with a DNA-fueled target recycling reaction was used for the isothermal, label-free, non-enzymatic amplification and ultra-sensitive electrochemical detection of nucleic acids [137]. Although it was only a proof-of-concept (depicted in Figure 5), the tandem could be exploited in environmental analysis of pathogens from aquatic ecosystems.

## 5. In-Situ Remote Sensing, HAB Monitoring in Buoys as Study Case

The Environmental Sample Processor is a fully autonomous, electromechanical fluidic system designed originally by the Monterey Bay Aquarium Research Institute (MBARI) to collect discrete water samples, concentrate particulates, and automate application of molecular analytical technologies [138]. It acquires and processes sample volumes of milliliters up to several liters at depths to 50 m. It is also capable of sub-surface deployments, but is generally co-deployed with contextual sensors that provide physico-chemical and biological data at the location and depth of the instrument. To provide a degree of passive mobility, the ESP has also been deployed on a drifter at a fixed depth and is amenable to shore-based/pier deployments.

It is commercially available from McLane Research Laboratories, Inc. (East Falmouth, MA, USA) but costs over $300,000 with its third generation currently being developed. Detection chemistries employ membrane-based DNA probe and protein arrays. A qPCR capability has been demonstrated for microbial targets [139]. DNA probe arrays target HAB species using probes designed from rRNA sequences in a SHA format for the simultaneous detection of multiple organisms in a single sample with chemilumiscent detection [134]. A competitive ELISA technique for detection of toxins produced by HAB species constitutes the protein arrays [140]. “The importance of immobilizing HAB detection onto autonomous platforms that can intelligently target sample acquisition as a function of environmental conditions and biological patch encounter” [141,142] is a goal that is paramount in all workers on toxic algal blooms.

## 6. Future Directions and Concluding Remarks

Conventional methods for pathogen analysis in aquatic ecosystems suffer from limitations and drawbacks when coming to practical applications. In this context, molecular techniques have step-by-step profiling as promising candidates for microorganisms monitoring in natural environments [143]. The practical utility of molecular-based techniques has shown to be limited by both the diversity of species and their presence in very low concentrations in environmental matrixes. Multiplexed biosensors, microarray formats, NGS or HTS have contributed to cover analysis of multiple species at the same time. Since the advent of nanomaterials, the development of nano-bioengineered probes and platforms have led to the development of biosensors of unprecedented features in terms of sensitivity, selectivity and detection limits. Magnetic separation systems offer an encouraging alternative for pre-concentration of low abundant genetic material. In the same manner, solid-phase hybridization is an option to capture low-abundance target DNA sequences. Some other approaches based on amplification of the target have been conducted to enhance the performance of molecular techniques. Whereas isothermal amplification allows for exponential amplification at a constant temperature, hybridization chain reaction provides a linear number of target copies in an enzyme-free isothermal format. In any case, the more important limitations of pathogen detection in environmental samples, in general, and of molecular techniques, in particular, are related to the time-consumption, costs, biodiversity and amount of genetic material required. Therefore, new methods that solve the aforementioned limitations must advance towards the development of high-throughput, cost-effective, more precise, sensitive and selective detection systems, with minimal power consumption, miniaturized size and portability compatible with outside and remote settings. Real time monitoring has only been achieved in one example from harmful algal blooms. However, the potential for many of these methods to make this final leap are well within the near future. Their use as an early warning system in any natural environment is laudable because their low cost and ease of implementation ensures that high frequency monitoring can take place to enable a rapid response time should any toxic or harmful organism in low abundance begin to increase.
